# Microcosm experimental evidence that habitat orientation affects phytoplankton-zooplankton dynamics

**DOI:** 10.1038/s41598-017-01618-2

**Published:** 2017-05-04

**Authors:** Yunshu Zhang, Ying Pan, Hanxiang Chen, Zhuomiao Hu, Shucun Sun

**Affiliations:** 1 Department of Biology, Nanjing University, 22 Hankou Road, Nanjing, 210093 China; 2 School of Ecology and Environmental Sciences, Yunnan University, Kunming, 650091 China; 3 Key Laboratory of Mountain Ecological Restoration and Bioresource Utilization & Ecological Restoration Biodiversity Conservation Key Laboratory of Sichuan Province, Chengdu Institute of Biology, Chinese Academy of Sciences, 9 Section 4 Renminnan Road, Chengdu, 610041 China

## Abstract

Although spatial ecology has achieved a great success in the passing decades, the importance of habitat orientation has not been well studied, especially for its effects on prey-predator dynamics. Here, we examined the responses of zooplankton activity and grazing rate to habitat orientation and their consequences on the stability of phytoplankton-zooplankton system in a two-factor factorial experiment involving habitat orientation (three levels; small, medium, and large base area, respectively) and habitat size (64 ml and 512 ml) using two algal-grazer systems (*Chlorella pyrenoidosa*-*Daphnia magna* and *C. pyrenoidosa*- *Moina micrura*). In both systems, grazer density increased with increasing base area for a given chamber volume and with increasing chamber volume for a given orientation in the first 6 days, followed by a dramatic decrease, which corresponded to increasing the amplitude of density fluctuations in both zooplankton and phytoplankton species. Such an algal-grazer dynamics could be accounted for by the greater average swimming ability and grazing rate observed in large-based and large-volumed chambers. Our results demonstrate that habitat orientation affects the zooplankton behavior and population dynamics of both zooplankton and phytoplankton species, which further influences the stability of phytoplankton-zooplankton systems.

## Introduction

In natural ecosystems, species habitats may be placed in space with different orientations. For example, either a two-dimensional (e.g. forest patches to bird species; but not a circular habitat) or three-dimensional (e.g. waters for aquatic species; but not a spherical habitat) habitat may be placed in space at different orientations (e.g. angles relative to light direction for two-dimensional habitats and different sides as chamber base for three-dimensional habitats, see Supplementary Fig. S1), even if the habitat size and shape hold constant. Habitat orientations are diverse in natural ecosystems, particularly under human-induced disturbances that often break up a single continuous habitat into several pieces of fragments.

Changes in habitat orientation can affect many environmental conditions (e.g. light, water current, heat, wind, nutrients, Earth’s gravity and magnetic field)^1, 2^ and thus might have consequences on various ecological processes. For example, van Kleunen & Fischer^3^ found that habitat orientation (relative to the light direction) plays an important role in determining the morphology of stoloniferous rosette species by varying light intensity in different directions. Community composition of migrating organisms can also be affected by habitat orientation (relative to their moving directions). Generally, habitats oriented perpendicular to the dispersal paths of migrating organisms intercept more species (birds or fishes) and individuals than comparable habitats oriented parallel to the line of travel in both terrestrial^2, 4^ and aquatic ecosystems^5^. However, the mechanisms underlying the habitat orientation effects have scarcely been explored, particularly in three-dimensional habitats.

Plankton systems are ideal for examining how habitat orientation affects ecological processes in three-dimensional habitats because of the close relationship between physical structure and plankton dynamics^6^. Habitat orientation (relative to the direction of gravity) may affect phytoplankton-zooplankton dynamics via physical processes^6, 7^. Firstly, varying habitat orientation may alter the distance from the base to the top of the space in the direction of gravity (i.e. vertical length), and thus will affect average swimming ability (as a result of changing energy costs in overcoming gravity) of herbivore grazers. Specifically, in cuboid chambers for a given volume and shape (e.g. constant ratio of length: width: height, see Supplementary Fig. S1), increasing base area will reduce energy costs in overcoming gravity and thus will increase the average swimming ability of zooplankton species^8, 9^. Consequently, habitat orientation may further affect the grazing rate of zooplanktons because the rate is often positively correlated with swimming ability^10, 11^. Increasing the grazing rate of zooplankton on phytoplankton cells will benefit the growth and reproduction of zooplankton grazers (as long as the availability of algae is unlimited) and lead to rapid growth in zooplankton numbers due to the close relationship among the growth, reproduction and grazing rate^12, 13^. Moreover, because of the close coupling between phytoplankton and zooplankton, increasing base area should ultimately cascade to reduce phytoplankton density, resulting in large population (density) fluctuations of both phytoplankton and zooplankton species and even zooplankton extinction following previous studies^14, 15^, i.e., will reduce the stability of phytoplankton-zooplankton system.

In one of our previous studies^9^, we have shown that habitat orientation affects swimming speed and foraging success for a single zooplankton species (*Ceriodaphnia quadrangular*) and for a very short time (24 hrs). This study is to test whether habitat orientation affects the long term (24 days) dynamics of phytoplankton- zooplankton systems (but not only individual performance). We conducted a microcosm experiment involving habitat orientation (three levels; placing cuboid chambers in three orientations with long, medium, and small side as the chamber height, respectively; see Supplementary Fig. S1) and habitat volumes (two levels; large vs. small) with two plankton systems (*Daphnia magna-Chlorella pyrenoidosa* and *Moina micrura-C. pyrenoidosa*). We investigated the population dynamics for both phytoplankton and zooplankton species in both systems. Moreover, to explore the mechanism underlying the orientation effect on the algal-grazer dynamics, we also conducted two independent short-term examinations to examine the effects of habitat orientation on the grazer swimming activity and grazing rate. As aforementioned, we hypothesized that: (1) average swimming ability, grazing rate, and the growth, survival and reproduction of grazers would be greater, and (2) the algal density would be lower in large-based chambers than in small-based ones at the early experimental stage, (3) which would finally reduce the stability of phytoplankton-zooplankton system in these chambers.

## Results

### Grazer behavior and grazing rate

Dissolved oxygen (DO) concentration was unaffected by habitat orientation and volume size (P > 0.4), and was always higher than 7.5 mg L^−1^ after 4 hours of cultivation in the grazing experiment (see Supplementary Table S1).

**Figure 1:**
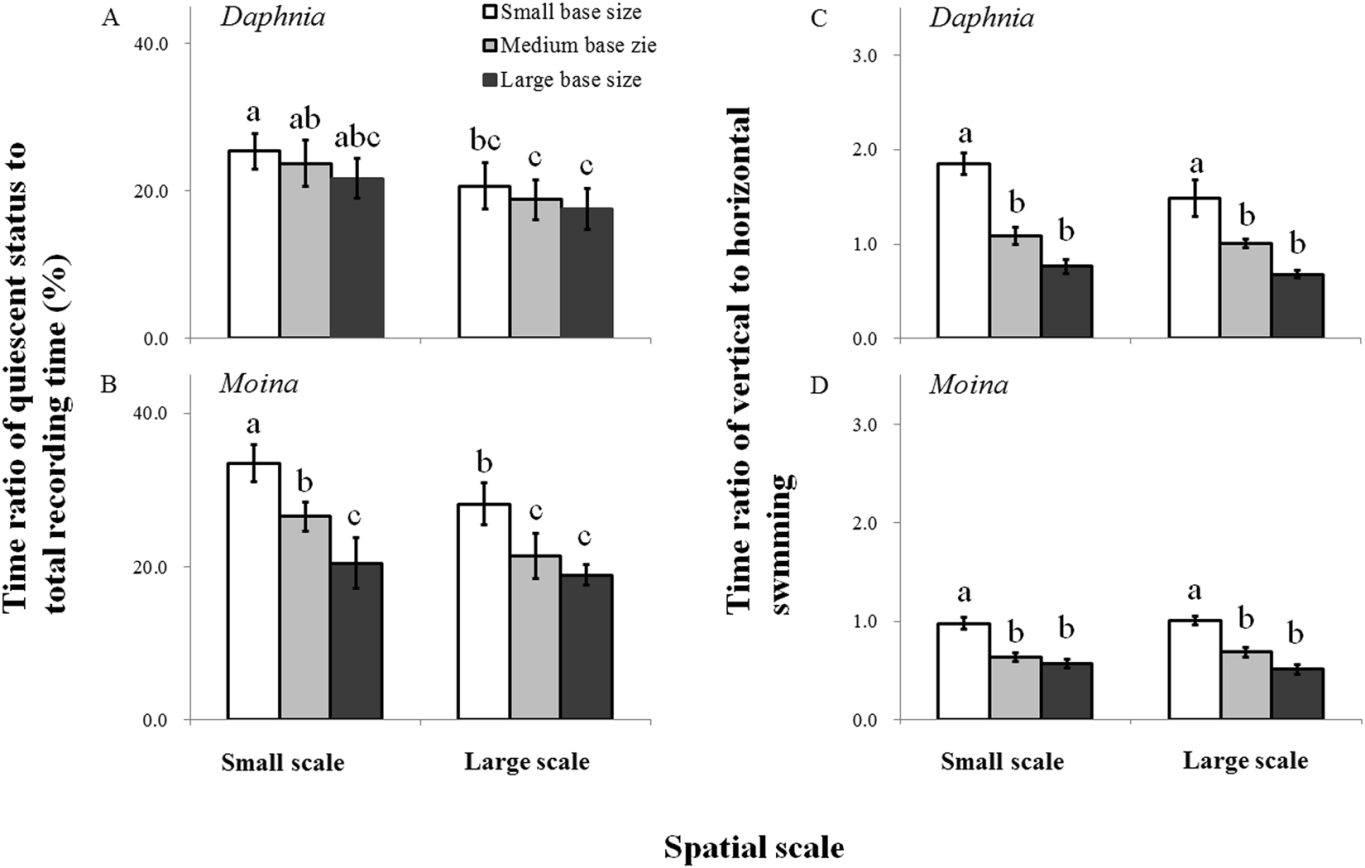
Time ratios (means ± 1 s.d., n = 6) of quiescent status to total recording time (**A**,**B**) and vertical to horizontal swimming (**C**,**D**) of grazer species (*Daphnia magna* and *Moina micrura* with the green alga *Chlorella pyrenoidosa* as the exclusive diet) under three levels of habitat orientation and two levels of spatial scale. Different letters indicate significant differences among treatments. Multiple comparisons of means were performed using Tukey’s test at the 0.05 significance level. Figure 1 was created using Microsoft Excel 2016.

**Figure 2:**
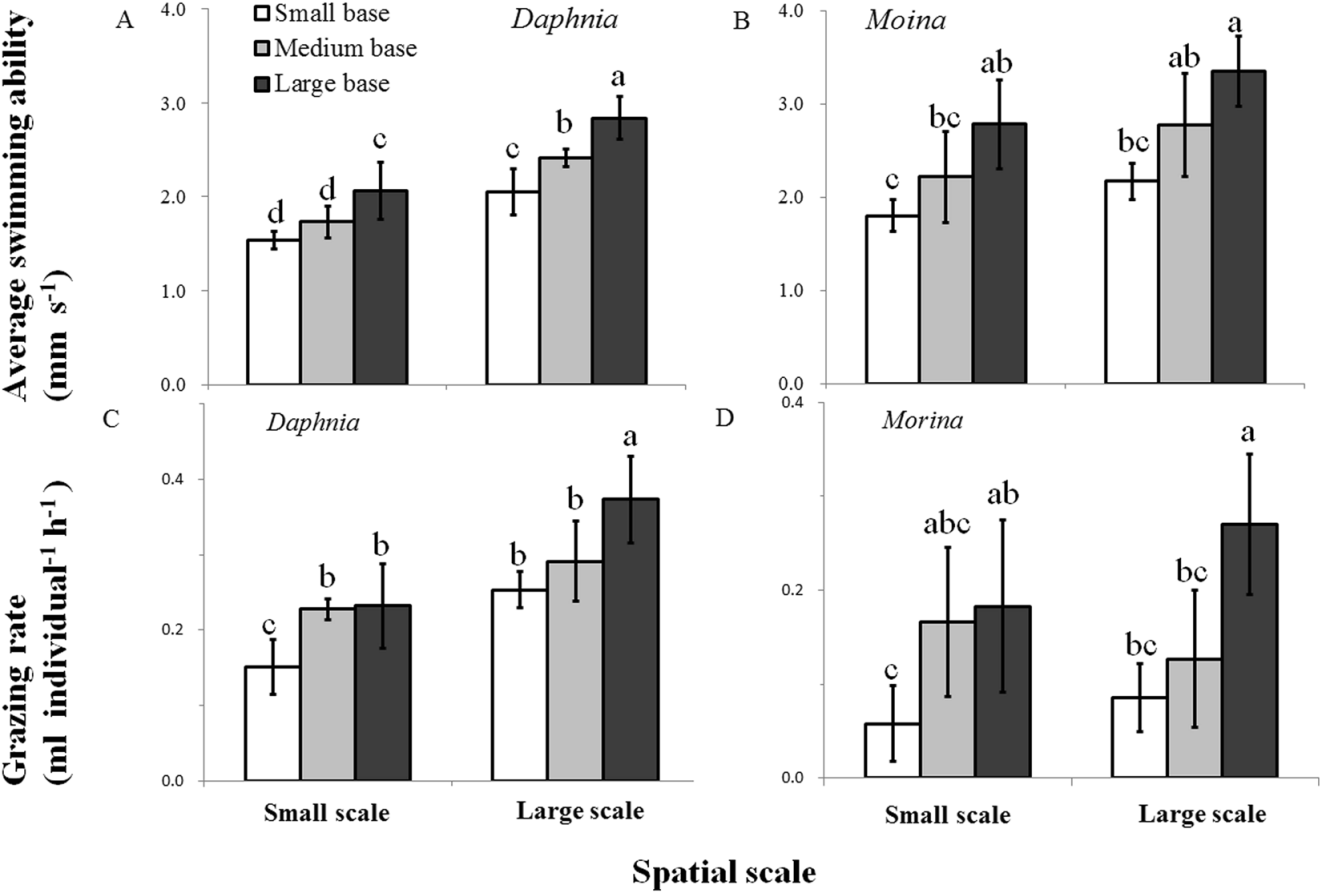
Average swimming ability (**A**,**B**) and grazing rate (**C**,**D**) of grazer species (*Daphnia magna* and *Moina micrura* with the green alga *Chlorella pyrenoidosa* as the exclusive diet) under three levels of habitat orientation and two levels of spatial scale (means ± 1 s.d., n = 6). Different letters indicate significant differences among treatments. Multiple comparisons of means were performed using Tukey’s test at the 0.05 significance level. Figure 2 was created using Microsoft Excel 2016.

In both plankton systems, swimming activity and average swimming ability were significantly affected by habitat orientation and volume size for grazer species (Figs 1 and 2A,B, Supplementary Tables S2 and S3). Specifically, with increasing base area for a given volume, the duration of quiescent status (Fig. 1A,B) and upward swimming (Supplementary Table S3) and the time ratio of vertical to horizontal swimming (Fig. 1C,D) decreased, but the velocity and duration (P < 0.001) of horizontal swimming increased (see Supplementary Table S3). The horizontal velocity was generally higher than that of upward swimming at all given chambers, and hence average swimming ability significantly increased with increasing base area (P < 0.001; Fig. 2A,B). For a given habitat orientation, increasing volume size led to increases in the duration and velocity of horizontal swimming but a decrease in the duration of quiescent status (especially for *D. magna*), which collectively increased the average swimming ability. Additionally, the positive effects of increasing volume size on average swimming ability in the grazer species was species-specific, which was more obvious for large species *D. magna* than small species *M. micrura* (Fig. 2A,B).

Variations in grazing rate were similar to those of the average swimming ability. In the presence of grazers, grazing rates of both *D. magna* and *M. micrura* consistently increased with increasing base area for a given chamber volume (P < 0.001; Fig. 2C,D). Besides, grazing rates of both grazer species generally increased with increasing volume size, especially for large species *D. magna* at small-based and large-based chambers (P < 0.05), as indicated by the significant volume size × grazer species interaction (P < 0.01, Supplementary Table S2). More evidently, the grazing rate was positively associated with average swimming ability in both algal-grazer systems (r^2^ ranging > 0.75; see Supplementary Fig. S2). However, in the absence of grazers, algal density of *C. pyrenoidosa* was unaffected by habitat orientation and volume size (P > 0.3; Supplementary Fig. S3).

### Algal-grazer dynamics

**Figure 3:**
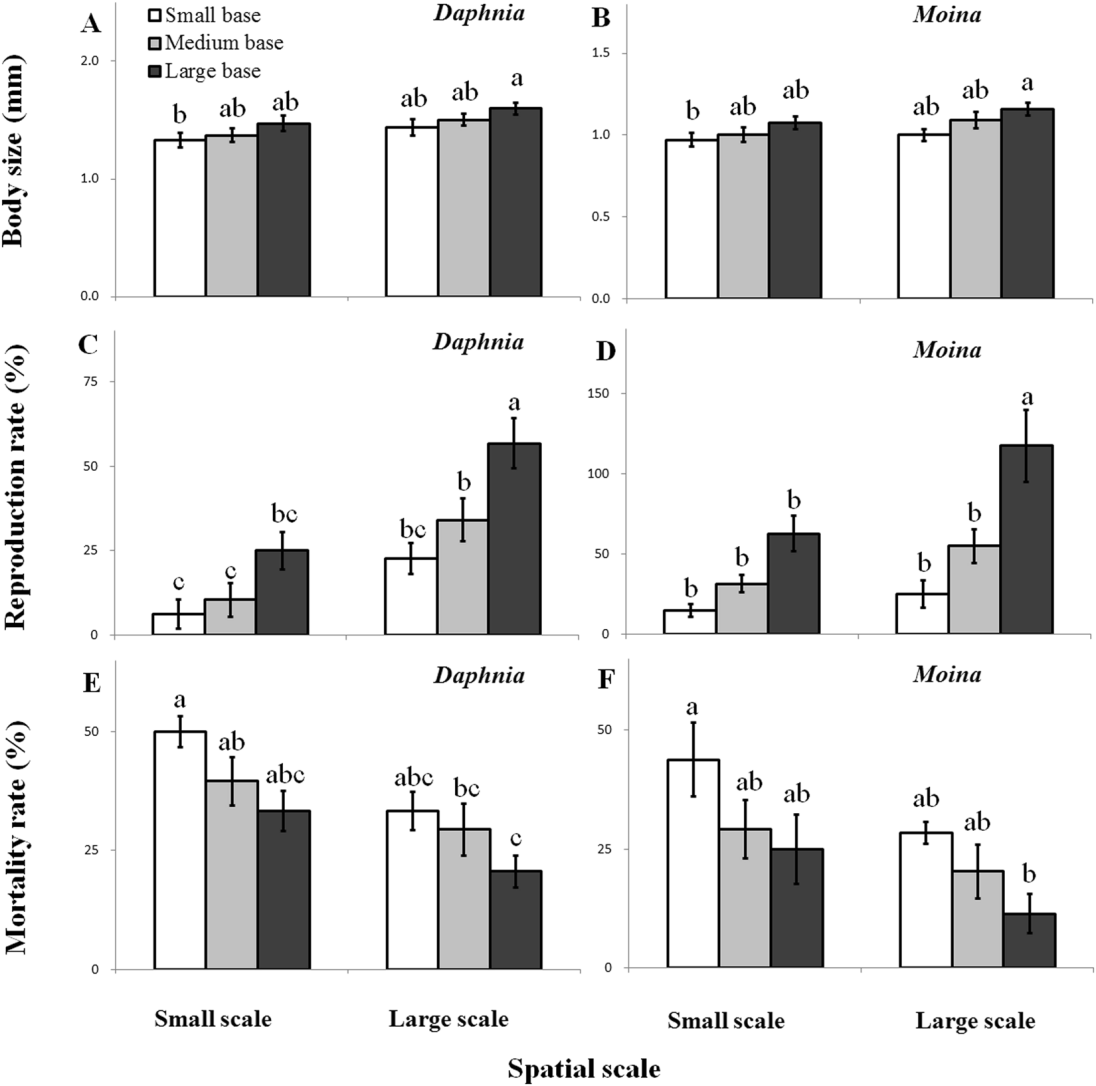
Variations (means ± 1 s.d., n = 6) in body size (**A**,**B**), reproduction rate (**C**,**D**) and mortality rate (**E**,**F**) of grazer species (*Daphnia magna* and *Moina micrura*) under three levels of habitat orientation and two levels of spatial scale. Different letters indicate significant differences among treatments. Multiple comparisons of means were performed using Tukey’s test at the 0.05 significance level. Figure 3 was created using Microsoft Excel 2016.

**Figure 4:**
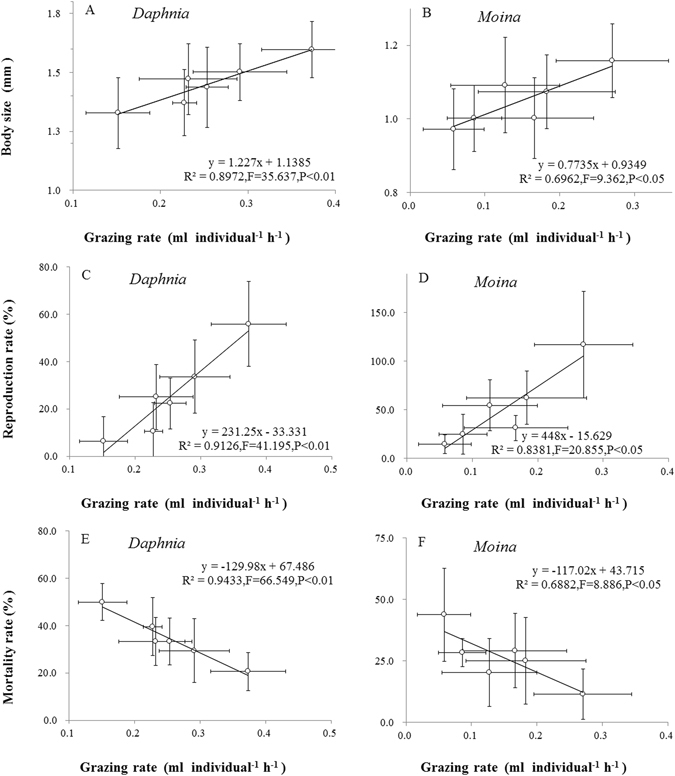
Linear regressions between grazing rate with body size, reproduction rate, and mortality rate of *Daphnia magna* and *Moina micrura* with green alga *Chlorella pyrenoidosa* as the exclusive diet under three levels of habitat orientation and two levels of spatial scale (n = 6). Figure 4 was created using Microsoft Excel 2016.

**Figure 5:**
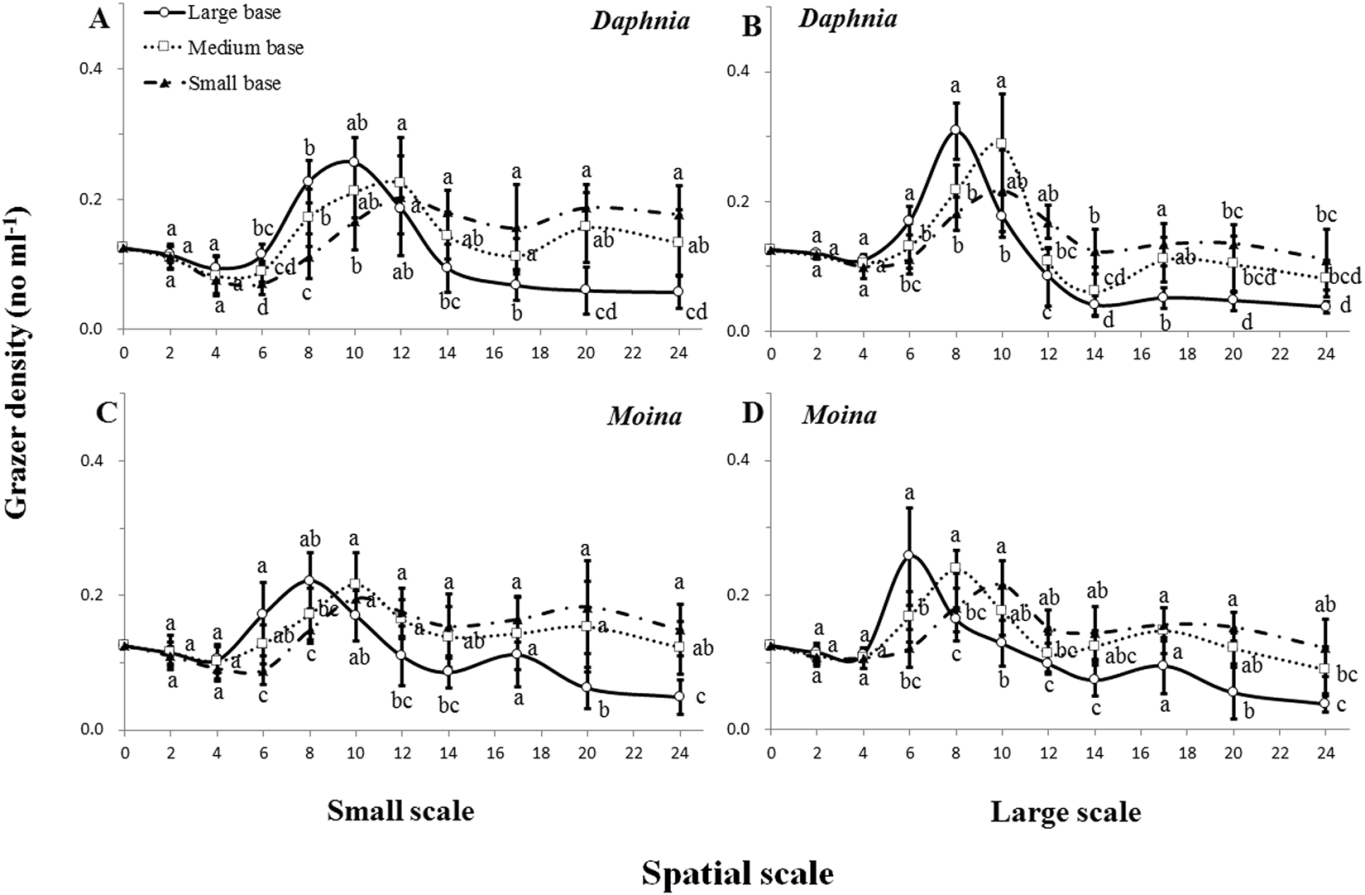
Grazer density (means ± 1 s.d., n = 6) of *Daphnia magna* (**A**,**B**) and *Moina micrura* (**C**,**D**), with green alga *Chlorella pyrenoidosa* as the exclusive diet, under three levels of habitat orientation and two levels of spatial scale. Different letters indicate significant differences among treatments on a specific day. Multiple comparisons of means were performed using Tukey’s test at the 0.05 significance level. Figure 5 was created using Microsoft Excel 2016.

**Figure 6:**
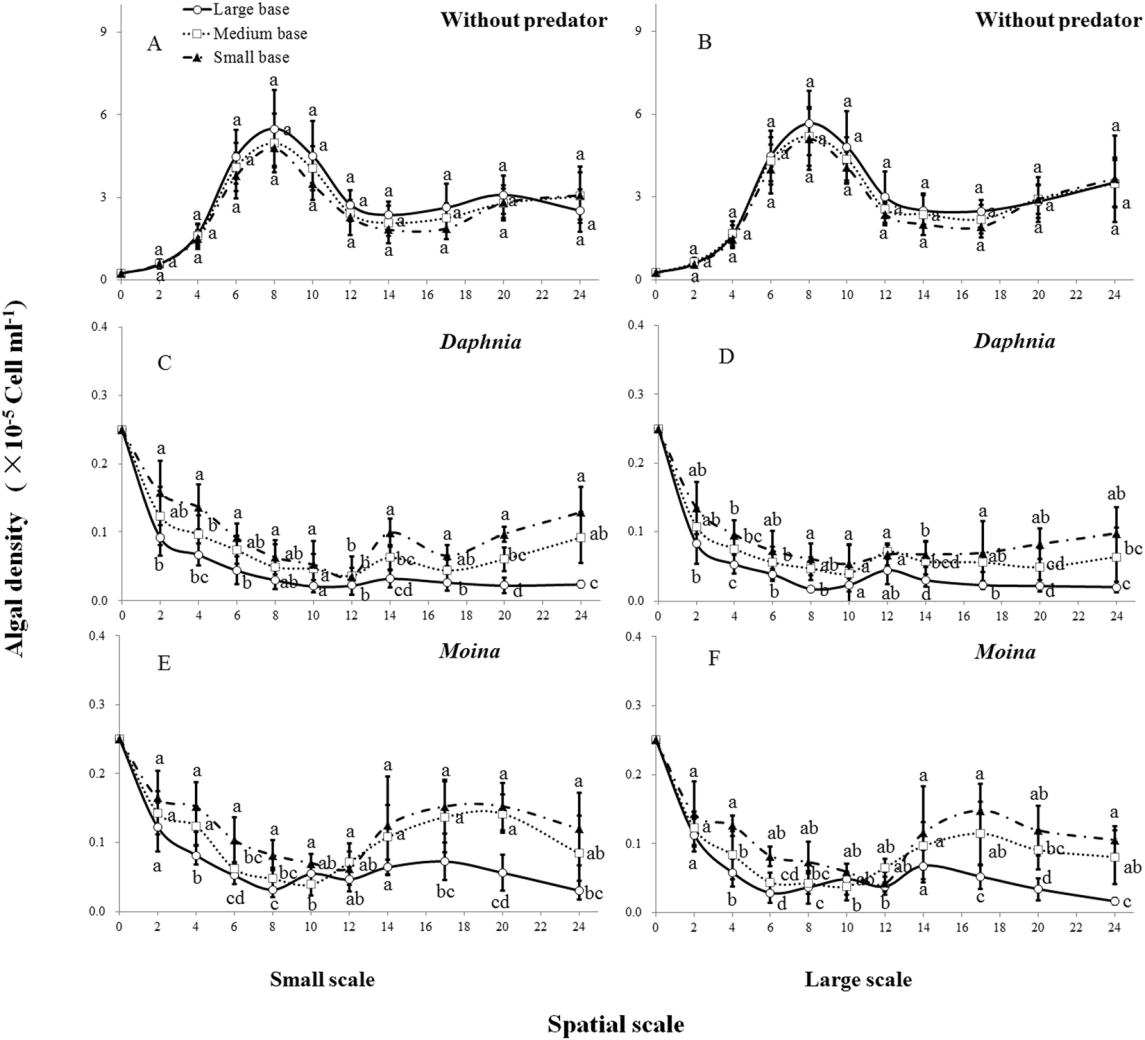
Cell density (means ± 1 s.d., n = 6) of *Chlorella pyrenoidosa* in the absence (**A,B**) and presence of grazers [*Daphnia magna* (**C**,**D**) or *Moina micrura* (**E,F**)] under three levels of habitat orientation and two levels of spatial scale. Different letters indicate significant differences among treatments on a specific day. Multiple comparisons of means were performed using Tukey’s test at the 0.05 significance level. Figure 5 was created using Microsoft Excel 2016.

In both plankton systems, population dynamics of grazer species were significantly affected by habitat orientation and volume size (Figs 3–6, Supplementary Table S4). After 6 days of culture, body size (Fig. 3A,B) and reproduction (Fig. 3C,D) of *D. magna* and *M. micrura* consistently increased, while mortality rate (Fig. 3E,F) generally decreased with increasing base area for a given chamber volume and with increasing chamber volume for a given orientation. A simple linear regression analysis showed that the grazing rate was significantly and positively correlated with body size (P < 0.05; r^2^ > 065; Fig. 4A,B) and reproduction rate (P < 0.05; r^2^ > 0.80; Fig. 4C,D), but negatively associated with mortality rate (P < 0.05; r^2^ > 0.65; Fig. 4E,F) after six days of culture.

Consequently, in both plankton systems grazer density in large-based chambers and large-volumed chambers quickly increased to a maximum at the early stage of the experiment (i.e. 6–10^th^ day) and the peak values were generally higher than those in small-based chambers and small chambers (Fig. 5A–D). Subsequently, grazer density rapidly declined and remained at a low level to the end of the experiment. At the end of the experiment, grazer density in large-based chambers decreased by 54.2% and 70.6% for *D. magna*, and by 60.4% and 69.8% for *M. micrura* of the original density, in small and large chambers, respectively. In contrast, grazer density in small-based chambers and small-volumed chambers initially lagged behind the formers, but subsequently increased and remained at a high level until the end of the experiment. Collectively, habitat orientation and volume size generally altered the timing of the density peak and its size in both grazer species, although the density of *M. micrura* was unaffected by volume throughout the whole experiment (Supplementary Table S4).

The algal density of *C. pyrenoidosa* was unaffected by habitat orientation and volume size on each observation day in the absence of grazers (P > 0.05; Fig. 6A,B). However, when the grazers were present, algal density consistently decreased with increasing base area for a given chamber volume in both plankton systems during the experiment (RM-ANOVA, P < 0.001; Supplementary Table S4). Specifically, cell densities of *C. pyrenoidosa* in chambers with large base sharply decreased to a minimum value at the early stage (8–10^th^ day for *D. magna* and 6–8^th^ day for *M. micrura*) and then remained at a consistently low level at the later stage of the experiment (Fig. 6C–F). In contrast, in chambers with small base, the decreases of cell density at the early stage of experiment were less obvious, and the algal density started to increase and remained at a high level in later experimental stages (i.e. 17–24^th^; P < 0.05). Generally, the production of primary producer generally decreased with increasing base area for a given chamber volume. Additionally, for a given orientation, large chambers tended to have a lower algal density than those of small chambers, particularly at the early stage of experiment (i.e. 2–6^th^ day).

## Discussion

We have shown that the densities of grazers and algae and the stability of the algal-grazer system were greatly affected by chamber orientation at both volumes, providing empirical evidence that habitat orientation affects prey-predator dynamics. Specifically, chamber orientation affected zooplankton swimming ability and grazing rate, which further influenced the growth, survival and reproduction of grazers, and subsequently affected algal and grazer density and the stability phytoplankton-zooplankton system in turn (see Supplementary Fig. S4). Provided that prey-predator dynamics have long been the dominant themes in community ecology^16^, we suggest that habitation orientation may be an important factor shaping species dynamics in natural systems.

### Orientation effects on swimming activity and grazing rate of grazers

Chamber orientation directly affected grazer activity by changing the physical regime within the chambers such as the vertical distance in the direction of gravity. Cladoceran individuals usually use a random searching strategy to maximize grazing rate^17, 18^. Such randomness should have occurred in the present study because differences in light conditions (light direction and intensity), DO concentration (Supplementary Table S1), cell density (Supplementary Table S5), and other factors that potentially affect grazer mobility have been intentionally minimized. Thus, increasing vertical length for a given volume led to increased duration of upward swimming and the time ratio of vertical to horizontal swimming in our experiment.

Cladoceran organisms generally have to spend more energy to swim upward the same distance as individuals swimming horizontally as a result of additional costs of overcoming gravity when Reynolds number >1^8, 19, 20^. Therefore, swimming velocity and swimming frequency of grazers were lower in chambers with long vertical length (small base area) than short ones, which might help maintain survival and growth^21–24^. As a result, average swimming ability was lower in chambers with small bases than those with large ones for a given chamber volume for both grazer species.

Lower swimming velocity is often more likely to decrease grazing rate because it decreases the encounter rate of a predator with its prey^25^. Consistently, average swimming ability was positively correlated with grazing rate in both plankton systems (Supplementary Fig. S2). Thus, it is not surprising that grazing rate was lower in chambers with small bases than those with large ones for a given chamber volume. In general, the responses of these two grazer were similar to that of *C. quadrangular* that was used in the previous study^8^.

### Orientation effects on the algal-grazer dynamics

Altered the grazer swimming activity and grazing rate may affect the dynamics of algal-grazer systems^26, 27^. Low grazing rate often limits body growth and reproduction, and can induce high mortality^13, 28, 29^. We observed that the mortality rate increased, and growth (body size) and reproduction decreased in both grazer species with decreasing base area for a given chamber volume, with significant relationships between grazing rate with body size, reproduction rate and mortality rate.

High grazing rate combined with quickly increased grazer density in large-based chambers, in turn, led to a rapid decline in the density of *C. pyrenoidosa*, which lacked effective strategies defending against cladoceran grazers. Consequently, the algal density in large-based chambers sharply decreased at the early experimental stage, which subsequently inhibited the growth and reproduction of grazer species, resulting in a steep decline in grazer density. Increasing the amplitude of density fluctuations in both zooplankton and phytoplankton species can reduce the stability of algal-grazer system according to previous studies^14, 15^ and also in this study. In contrast, in small-based chambers, growth and reproduction of grazer species initially lagged behind those in large-based chambers, allowing a relatively high algal density at the early experimental stage, such that the grazer population was not largely reduced in later stages. At the end of the experiment, the densities of both algae and grazer were much lower in the large-based than small-based chambers regardless of volume size and plankton system. In conclusion, orientation altered the density and fluctuation of both algal and grazer populations and the stability of algal-grazer system.

It is worthwhile to note that orientation effect on population dynamics of *C. pyrenoidosa* in the absence of grazers (Supplementary Table S4) does not invalidate the conclusion that habitat orientation significantly affected algal-grazer dynamics. Although increasing base area led to an increase in algal density in the absence of grazers, the presence of grazers led to significantly decreased algal density with increasing base area. This exactly indicates an orientation-improved grazing rate in large-based chambers compared with the small-based chambers.

In addition, we have also clearly shown that volume size also played an important role in modulating algal-grazer dynamics in both plankton systems, as noted by studies addressing spatial scale-dependent species interactions^30, 31^. This volume-size effect could be inferred from the comparisons between chambers with different volumes but the same orientation. Studies have shown that swimming behavior of zooplankton individuals greatly depends on the scale of habitats. For example, Dodson *et al*.^32^ and Dur *et al*.^33^ found that increasing chamber volume significantly increased the swimming velocity and frequency of zooplankton species. Consistently, increasing the chamber volume improved the swimming of grazer individuals (as indicated by shortened duration of quiescent status and increased duration and velocity of horizontal swimming), and thereby increasing average swimming ability and grazing rate in this study, which further accelerated the population growth rate of grazer species and delayed the increase of algal population. As a result, the stability of algal-grazer system was lower in large-volumed chambers than in small-volumed chambers. Nevertheless, the volume-size effect was more obvious for large species *D. magna* than small species *M. micrura*, although the mechanism underlying the difference has not been explored.

## Summary

Our results show that both habitat orientation and spatial scale can affect species interactions by changing species behavior, growth and reproduction, which further affect species population dynamics. We have not only provided empirical evidence that habitat orientation affects prey-predator dynamics but also revealed the underlying mechanisms. Moreover, our results may have implications for prey-predator dynamics in natural aquatic systems. In small natural ponds, the behavior and foraging efficiency of zooplankton grazers should be shaped by habitat orientation, thereby leading changes in population density of both zooplankton and phytoplankton. Similarly, in large aquatic systems, population dynamics of large animals may be also shaped by habitat orientation^34^. For instance, small tadpoles preferring small shallow ponds to deep ones, as well as many fishes preferring shallow to deep waters, could be partly due to the increased energy cost for foraging in deep waters^34, 35^. Given that some zooplankton species (e.g. copepods) has evolved an adaptive behavioural mechanism to retain swimming efficiency in turbulent flows^36^, the effects of habitat orientation on population dynamics of algae and grazer would not be neglected as well in constantly flowing environments. In addition, our results also indicate that habitat orientation should be considered for chamber microcosm studies addressing phytoplankton-zooplankton dynamics.

## Methods

### Experimental organisms

Green alga *Chlorella pyrenoidosa* (FACHB-28) was obtained from the Freshwater Algae Culture Collection of the Institute of Hydrobiology, the Chinese Academy of Sciences. This algal species is common in many ponds and lakes in China, and is preferred by many cladoceran species including *Daphnia* and *Moina* spp.^37, 38^. Algae were cultured aseptically in 250-mL Erlenmeyer flasks containing 100 mL of COMBO medium in an incubator with controlled temperature (20 °C), photoperiod regime (14 h^L^:10 h^D^), and light levels (50 μmol photons m^−2^ s^−1^). *C. pyrenoidosa* was maintained in exponential growth phase by periodic dilution with fresh medium.

Two cladoceran species (*Daphnia magna* and *Moina micrura*) were collected from Lake Taihu (31.5°N, 120.1°E). Both species play important roles in regulation of phytoplankton biomass in aquatic environments^39^. We cultivated monoclonal groups of the two cladoceran species in COMBO medium, and fed them with *C. pyrenoidosa* at a rate of 10^5^ cells mL^−1^ day^−1^ for over three months prior to the experiment.

Prior to the experiments, the grazers (2 to 3 days old) were transferred to clean COMBO medium and starved for at least 4 hours. Body size of *D. magna* and *M. micrura* ranged from 0.65 to 1.7 mm and from 0.5 to 1.16 mm in our early cultures, respectively, and we chose the medium-sized individuals of each grazer species (about 1.1 mm long for *D. magna* and 0.83 mm for *M. micrura*) as experimental grazers because large individuals might bear embryos and reproduce during experiments and small individuals would have small Reynolds numbers (<1) according to our previous studies^8, 9^.

### Experimental design

A two-way factorial design was applied that combined three levels of habitat orientation (differing in base area) and two levels of spatial scale (64 mL and 512 mL in volume), resulting in six treatments (see Supplementary Fig. S1), with each treatment having 18 replicates (cuboid transparent polyethylene containers). The side length ratio of each chamber was 1:2:4. The 18 cuboid chambers were used for three different experiments on the activity, grazing rate, and algal-grazer dynamics, with each experiment having six replicate chambers. Each experiment was replicated twice for both *C. pyrenoidosa*-*D. magna* and *C. pyrenoidosa*-*M. micrura* systems. Two additional treatments included only algal species were also set for each of the above treatments in the grazing experiment and algal-grazer dynamics experiment, so as to accurately estimate the grazing rate of the two grazer species and the population dynamic of *C. pyrenoidosa* in the absence of grazers. Each of such additional treatments had six replicates.

The experimental volume was intentionally set at a small scale to presumably minimize physic-chemical differences across the water column. Nevertheless, the volume was within the range used in many previous works addressing grazer activities and algal-grazer interactions^40–42^. The initial grazer densities in all treatments were 125 individuals L^−1^, which was similar to previous studies addressing cladoceran activities and species interactions^43, 44^, and was within the range observed in natural environments^45^. The initial algal densities in all treatments were approximately 2.5 × 10^4^ cells mL^−1^, which has been used by many other microcosm experiments^15, 46^. All the beakers were transferred to incubators (TS-2102GZ, Shanghai Anjing laboratory equipment Co., Ltd) that were set at 20 °C with light intensity being about 50 μmol photons m^−2^ s^−1^. Both behavioral and grazing experiments were conducted within a day from 8:00 am until 5:00 pm on 12-Feb-2014 (Beijing time).

### Behavioral experiment

Experiment was conducted at 20 °C in laboratory. After the beakers were mechanically stirred at 60 rpm for 5 minutes to homogenize the algal distribution in the beakers, the beakers were taken out the incubators, and then the grazers were allowed to acclimatize to the new environment for 15 minutes. Standard fluorescent bulbs were installed approximately 2 m away around (including the top and all sides) the experimental setup providing illumination of about 40 μmol photons m^−2^ s^−1^. Such a physical setting may not only minimize the grazer reaction of positive phototaxis that confounds the random-walk pattern of zooplankton grazers, but also allow for our results applicable to the animals that do not show phototaxis in aquatic systems^9, 17, 18^.

Swimming activities of grazer individuals were recorded with two cameras (see Supplementary Fig. S5). The two video cameras (GZ-VX855BAC, JVC, Japan) were placed orthogonally at a distance of 26 cm from the central of the projective plane (i.e., the bottom or the backside of the chamber). This physical setting allowed for concurrent recording of the swimming activity of a grazer individual in both horizontal and vertical directions. Each experimental chamber was recorded for 5 min at 60 frame s^−1^ with a resolution of 1920 × 1080 pixels^8, 9, 42^.

Video tapes were reviewed using an image measurement tool (Adobe After Effects CS4). Four different types of activities were recorded for one randomly-chosen grazer individual following Buchanan *et al*.^47^ and Gorski & Dodson^48^: horizontal swimming, upward swimming, downward swimming, and quiescent status. First, we recorded the time for the four types of activities. Then we chose the fragments that contained swimming trajectories away from the walls of the chamber and were longer than 2 s^48, 49^ to calculated the instantaneous swimming velocity as the distance swum by this grazer individual between two frames (i.e. 16.9 ms) using ImageJ 1.46 and MTrackJ plugin^42, 50^. Among these metrics, the duration and distance of horizontal movement were measured from the camera in the vertical axes, while metrics about vertical movement and immobile motionless were measured from the camera in the horizontal axis. While analyzing the swimming velocity of an individual, each video was first calibrated to convert pixels into real distances (mm) using reference marks. Finally, the average swimming ability (*V*_*average*_, mm s^−1^) for the chosen grazer individual was approximated as followers:

1 $${V}_{average}=({V}_{h}{T}_{h}+{V}_{u}{T}_{u}+{V}_{d}{T}_{d})/({T}_{h}+{T}_{u}+{T}_{d}+{T}_{q})$$

where *T*_*h*_, *T*_*u*_, *T*_*d*_ and *T*_*q*_ are the durations of horizontal swimming, upward swimming, downward swimming and quiescence (s), respectively; *V*_*h*_, *V*_*u*_, and *V*_*d*_ are the velocities of horizontal swimming, upward swimming, and downward swimming (mm s^−1^).

Three animals were randomly chosen to determine the above mentioned metrics in each chamber following previous studies^8, 9, 51^. A total of 108 grazer individuals (3 individuals per chamber × 6 chambers per treatment × 6 treatments) were followed in the behavioral experiment. These metrics of each grazer individual obtained during behavioral investigation was first averaged for each beaker and then for each treatment.

### Grazing experiment

Investigation of grazing rate was run in the incubators. Each chamber was capped with breathable polyethylene films, mechanically stirred (at 60 rpm) and gently aerated with sterile filtered air (Sartorius, Midisart 2000) for 5 min every 1 hour to homogenize dissolved oxygen (DO) concentration and algal density. An independent examination (containing algae only) showed that algal density was indistinguishable between the top and bottom layers of the experimental chambers within one hour (see Supplementary Table S5) after a stirring event presumably because of low sinking rate of the small-sized *C. pyrenoidosa* cells^52, 53^. Algae were sampled 4 hours after the beginning of the experiment. For each sampling, we first measured DO concentration using a Hach HQ40d oxygen probe (Hach, Loveland, Colorado, USA). Then, 2 mL of sampled solution was removed to a 10 mL tube containing 0.1 mL of Lugol’s fixative for the microscopic enumeration of algal cells. At the end of the experiment, algal density was determined using a light-microscope at 400x magnification.

The grazing rate of cladoceran grazers (*G*, mL animal individual^−1^ h^−1^) was calculated as the difference in algal density between the experimental treatments (with grazer) and the corresponding controls (without grazers) according to the commonly used equation for planktons^54^, i.e.

2 $$G=V\times [{\rm{In}}\,({C}_{{0}}/{C}_{{1}})]/NT$$

where *V* is the chamber volume (mL); *C*_*0*_ and *C*_*1*_ are the algae density at the end of the measurement in the control and experimental chambers (cell mL^−1^), respectively, *N* is the number of the grazer individuals within the chamber, and *T* is the duration of the experiment (4 h).

### Algal-grazer dynamics experiment

This experiment was designed to evaluate whether habitat orientation affected the dynamics of algal-grazer populations. Experiment was carried out in incubators that were controlled under the same conditions as for the investigation of grazing rate, except for using a light-dark cycle of 14 h:10 h. The growth of algae and grazer were measured on the days 2, 4, 6, 8, 10, 12, 14, 17, 20, and 24 at 8:00 after the treatments were initiated. On each sampling day, we took each beaker out of the incubator and removed 5% of the volume, and 2 mL of these removed solutions was transferred into a 10 mL tube that contained Lugol’s preservative for the measurement of *C. pyrenoidosa*. Then, 5% volume of fresh medium was added to each beaker to replenish nutrients and prevent metabolic waste build-up. Subsequently, each beaker was capped and replaced back into the incubator.

In additional to counting algae, we measured the body size of five randomly chosen adult females, and the reproduction rate and mortality rate of cladoceran grazer in each chamber on day 6, when the size differences between the parental and neonatal grazers were most obvious and when the grazers were at the stage before first-generation offspring began producing new neonates. Body size was determined as the length from top of the head to the tip of the abdomen, which was measured using a light-microscope at 40x magnification. Reproduction rate and mortality rate was estimated as the number of offspring and dead adults to the total number of individuals added at the start of the experiment, respectively. After measurement, the grazers were placed back into the beakers.

### Data analysis

All data were tested for normality and variance heterogeneity prior to analyses. Three- and two-way ANOVAs were used to determine the effects of habitat orientation, volume size, and grazer species on the average swimming ability and the grazing rate, and to determine the effect of habitat orientation and volume size on the DO concentration, swimming activity, average swimming ability and grazing rate of the grazers, and population dynamics of grazer (including grazer density, body size, reproductive rate and mortality rate) and algae (cell density) on each observation day, respectively, followed by Tukey’s *post hoc* test once a significant effect was detected. Two-way repeated measures ANOVA (RM-ANOVA) followed by a Bonferroni *post hoc* test were used to test the effects of habitat orientation and volume size on population dynamics (density) of algae and grazer. The sphericity assumption was evaluated with the Mauchly’s test, and in the case of violation the Greenhouse-Geisser correction was applied to recalculate the *F*-value. In addition, linear regression analyses were conducted to determine the relationship between the average swimming ability and grazing rate and between the grazing rate with body size, reproduction rate and mortality rate for both algal-grazer systems. All analyses were carried out using the IBM SPSS19.0 package (SPSS Inc., USA).

## Electronic supplementary material

Supplementary material
