# Supplementary material for: Microcosm experimental evidence that habitat orientation affects phytoplankton-zooplankton dynamics
Source: Sci Rep. 2017 May 4;7:1443. doi: 10.1038/s41598-017-01618-2 (PMC5431447; doi:10.1038/s41598-017-01618-2)
Supplement: Supplementary file 1 — Supplementary material [file 41598_2017_1618_MOESM1_ESM.pdf]

Microcosm experimental evidence that habitat orientation affects phytoplankton-zooplankton dynamics

Yunshu Zhang[1†], Ying Pan[1,2†], Hanxiang Chen[1], Zhuomiao Hu[1], Shucun Sun[1,3*]

[1]Department of Biology, Nanjing University, 22 Hankou Road, Nanjing 210093, China

[2]School of Ecology and Environmental Sciences, Yunnan University, Kunming 650091, China

[3] Key Laboratory of Mountain Ecological Restoration and Bioresource Utilization & Ecological Restoration Biodiversity Conservation Key Laboratory of Sichuan Province, Chengdu Institute of Biology, Chinese Academy of Sciences, 9 Section 4 Renminnan Road, Chengdu 610041, China

[†] These authors contributed equally to this work

*Corresponding author: shcs@nju.edu.cn

Supplementary Fig. S1 Illustration showing three levels of habitat orientation and two levels of spatial scale. H: the average distance along the direction of gravity. The side length ratio was 1:2:4 for each chamber.

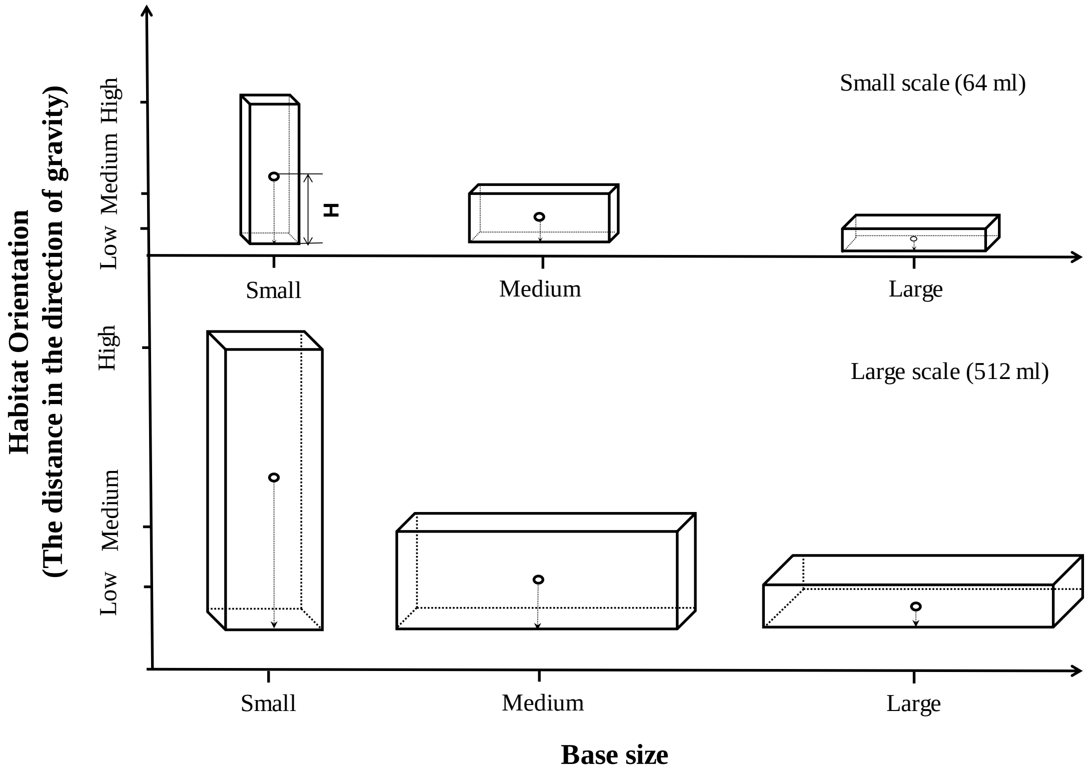

Supplementary Fig. S2 Linear regressions between average swimming ability and grazing rate of *Daphnia magna* and *Moina micrura* with green alga *Chlorella pyrenoidosa* as the exclusive diet under three levels of habitat orientation and two levels of spatial scale (n = 6).

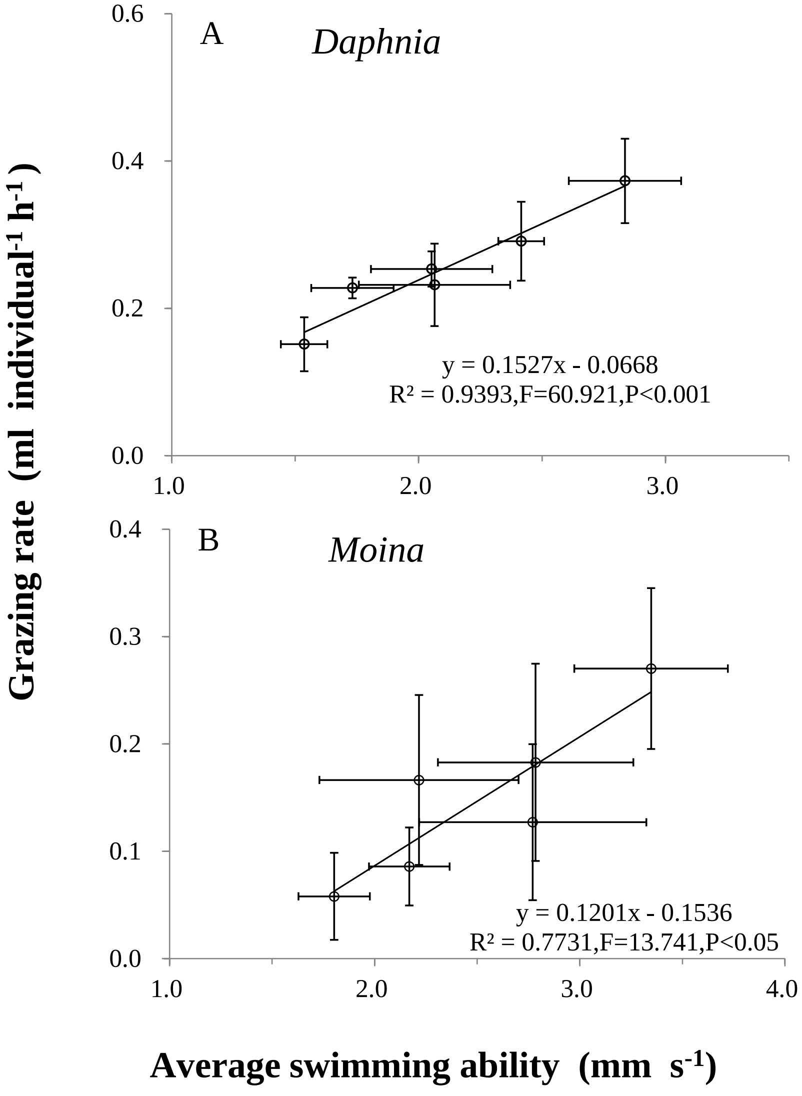

Supplementary Fig. S3 Algal density (means ± s. d, n=6) of *Chlorella pyrenoidosa* in the absence of grazer species under three levels of habitat orientation and two levels of spatial scales.

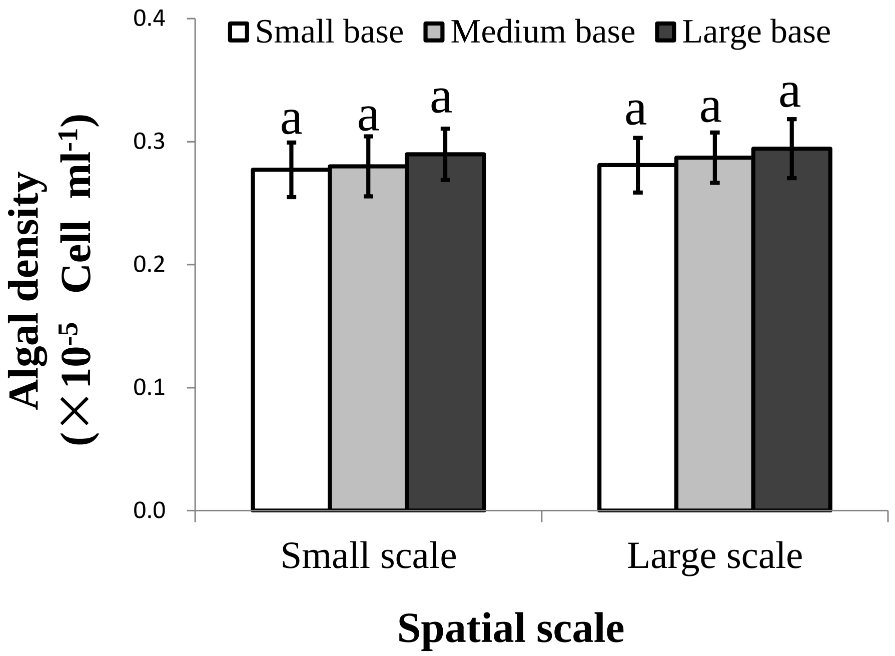

Supplementary Fig. S4 A conceptual figure of the effects of habitat orientation in affecting phytoplankton-zooplankton dynamics.

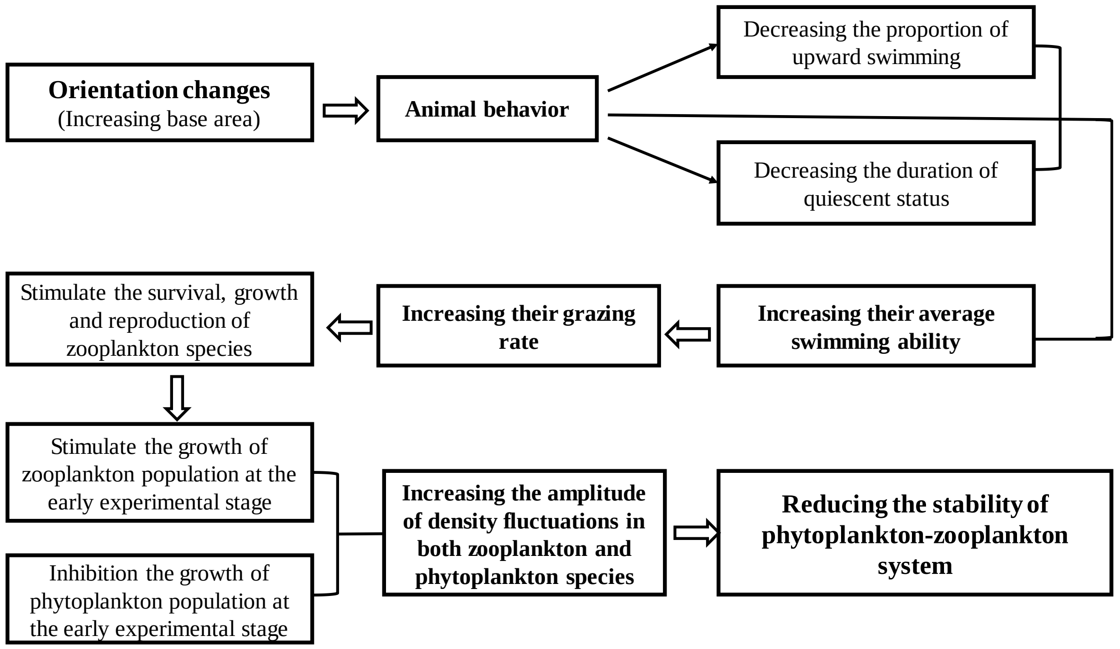

Supplementary Fig. S5

Schematic representation of the data acquisition system. The cladoceran individuals are kept in a cubic transparent polyethylene chamber and its swimming pathway is recorded using two synchronized and orthogonally focused cameras.

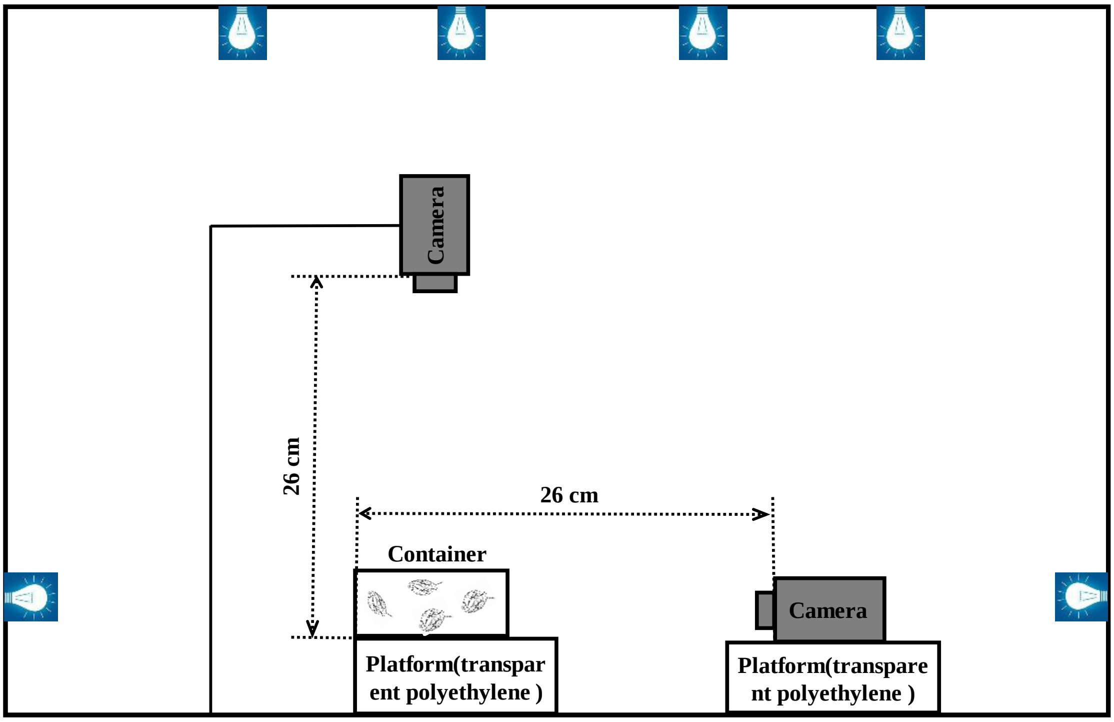

Supplementary Table S1 Dissolved oxygen concentration (means ± s. d., n = 6) in two plankton systems (*Chlorella pyrenoidosa*-*Daphnia magna* and *C. pyrenoidosa*-*Moina micrura*) under different spatial conditions after 4 hours of cultivation in the grazing experiment. Different letters indicate significant differences among treatments. Multiple comparisons of means were performed using Tukey's test at the 0.05 significance level.

		Control	*Daphnia magna*	*Moina micrura*
Small scale	Small base area	8.18±0.41a	8.07±0.40a	8.14±0.62a
	Medium base area	8.11±0.67a	8.11±0.51a	8.07±0.56a
	Large base area	8.14±0.56a	7.95±0.40a	7.94±0.52a
Large scale	Small base area	8.23±0.30a	8.12±0.58a	8.14±0.57a
	Medium base area	8.09±0.67a	7.77±0.50a	7.87±0.38a
	Large base area	8.01±0.50a	7.86±0.48a	8.12±0.40a

Supplementary Table S2 Summary of three-way ANOVA determining effects of habitat orientation, spatial scale and grazer species on average swimming ability and grazing rate (n = 6) for the whole dataset pooled from all short-time experiments with two different systems containing grazers.

	df	Average swimming ability (mm s^{-1})	Grazing rate (mL $individual^{-1}$ h^{-1})
Orientation (O)	2	43.812***	29.172***
Spatial scale (S)	1	64.420***	21.172***
Grazer species (G)	2	27.947***	61.125***
O×S	2	0.177^{ns}	4.712*
O×G	4	1.190^{ns}	1.403^{ns}
S×G	2	3.138^{ns}	8.013**
O×S×G	4	0.027^{ns}	0.274^{ns}

^{ns} P > 0.05; * P < 0.05; ** P < 0.01; *** P < 0.001

Supplementary Table S3 Swimming activity (means ± s.d., n = 6) of *Daphnia magna* and *Moina micrura* in different algal-grazer systems under three levels of habitat orientation and two levels of spatial scales. Different letters indicate significant differences among treatments. Multiple comparisons of means were performed using Tukey's test at the 0.05 significance level.

		Time distribution (s)				Swimming velocity (mm/s)		
		Quiescent status	Horizontal move	Upward swimming	Downward swimming	Horizontal move	Upward swimming	Downward swimming
Daphnia magna	SS	76.2±7.3a	98.7±12.1e	134.6±15.9a	42.9±10.7a	1.96±0.24c	1.69±0.28a	2.75±0.41a
	SM	71.3±9.3ab	132.5±8.0cd	93.0±10.4bc	47.4±11.5a	2.22±0.30c	1.84±0.31a	2.78±0.65a
	SL	65.2±8.0abc	157.2±11.2a	74.5±11.3c	43.2±11.3a	2.84±0.55b	2.07±0.41a	2.39±0.23a
	LS	62.1±9.6bc	121.5±16.5d	120.5±11.8a	49.0±8.1a	2.93±0.35b	1.85±0.50a	2.91±0.51a
	LM	56.6±8.1c	145.9±7.8bc	94.7±9.7b	49.4±9.2a	3.55±0.33a	1.77±0.51a	3.07±0.84a
	LL	52.7±8.2c	174.1±14.3a	77.2±11.9bc	38.0±5.9a	3.82±0.41a	2.37±0.51a	3.45±0.99a
Moina micrura	SS	100.4±7.2a	124.3±10.8c	84.2±7.6ab	34.0±13.9a	2.53±0.54b	2.24±0.22a	3.37±0.72a
	SM	79.6±5.8b	161.6±10.8b	73.0±10.8bc	27.4±2.6a	2.95±0.98b	2.41±0.16a	3.73±0.28a
	SL	61.4±10.0c	184.1±17.5a	70.1±10.9bc	32.7±6.3a	3.76±0.56ab	2.27±0.47a	3.48±0.79a
	LS	84.6±8.1b	132.5±10.2c	92.1±8.2a	39.5±4.3a	2.89±0.52b	2.41±0.21a	3.73±0.43a
	LM	64.1±8.8c	163±10.4b	78.5±11.7abc	32.8±11.1a	3.60±1.04ab	2.58±0.21a	4.49±0.71a
	LL	56.8±4.2c	192.2±13.0a	65.1±14.6c	30.8±1.5a	4.44±0.49a	2.53±0.45a	4.45±1.18a

Supplementary Table S4 Two-way repeated measures ANOVA results for the effects of time, habitat orientation and spatial scale on algal density and grazer density (n=6) in different algal-grazer systems.

	Time (T)	Orientation (O)	Scale (S)	T×O	T×S	O×S	T×O×S
Algal density in the absence of grazers (cell mL^{-1})	142.604***	3.617*	2.225^{ns}	0.509^{ns}	0.951^{ns}	0.019^{ns}	0.140^{ns}
Algal density in the presence of *Daphnia magna*	35.044***	163.165***	17.415***	2.378^{ns}	3.514*	1.780^{ns}	0.362^{ns}
Algal density in the presence of *Moina micrura*	33.021***	123.686***	30.277***	3.921*	1.097^{ns}	0.697^{ns}	0.206^{ns}
Grazer density of *D. magna* (no mL^{-1})	60.195***	8.218**	4.340*	15.178***	17.701***	0.302^{ns}	2.889^{ns}
Grazer density of *M. micrura*	30.552***	25.581***	1.455^{ns}	11.727***	4.453*	0.667^{ns}	1.724^{ns}

^{ns} P > 0.05; * P < 0.05; ** P < 0.01; *** P < 0.001

Supplementary Table S5 Densities (means ± s.d., n = 6) of *Chlorella pyrenoidosa* (for the experiment with algae only) across different layers of the water column in large-volumed and small-based experimental chambers. The densities of alga were monitored at the 1st, 2nd, 3rd hour in the experiment with a frequency of 5 min stirring per hour (see more details in text). Different letters indicate significant differences among treatments. Multiple comparisons of means were performed using Tukey's test at the 0.05 significance level.

		1 hour	2 hour	3 hour
Cell density ($\times 10^{-5}$ cell ml^{-1})	Bottom layer (12-16 cm)	0.254±0.03a	0.248±0.036a	0.274±0.04a
	Middle layer (6-10 cm)	0.252±0.028a	0.249±0.041a	0.261±0.044a
	Top layer (0-4 cm)	0.256±0.044a	0.245±0.039a	0.256±0.039a
